# Neuronal extracellular vesicle derived miR-98 prevents salvageable neurons from microglial phagocytosis in acute ischemic stroke

**DOI:** 10.1038/s41419-020-03310-2

**Published:** 2021-01-06

**Authors:** Jin Yang, Lu-Lu Cao, Xi-Peng Wang, Wei Guo, Ruo-Bing Guo, Yu-Qin Sun, Teng-Fei Xue, Zhen-Yu Cai, Juan Ji, Hong Cheng, Xiu-Lan Sun

**Affiliations:** 1 Department of Pharmacology, Neuroprotective Drug Discovery Key Laboratory, Jiangsu Key Laboratory of Neurodegeneration, Center for Global Health, Nanjing Medical University, Nanjing, China; 2 Zhongda Hospital, Southeast University, Nanjing, China; 3 The First Affiliated Hospital of Nanjing Medical University, Nanjing, China; 4 Nanjing University of Chinese Medicine, the Affiliated Hospital of Nanjing University of Chinese Medicine, Nanjing, China

**Keywords:** Stroke, Stroke

## Abstract

Extracellular vesicles (EVs), as a novel intercellular communication carrier transferring cargo microRNAs (miRNAs), could play important roles in the brain remodeling process after ischemic stroke. However, the detailed mechanisms involved in EVs derived miRNAs-mediated cellular interactions in the brain remain unclear. Several studies indicated that microRNA-98 (miR-98) might participate in the pathogenesis of ischemic stroke. Here, we showed that expression of miR-98 in penumbra field kept up on the first day but dropped sharply on the 3rd day after ischemic stroke in rats, indicating that miR-98 could function as an endogenous protective factor post-ischemia. Overexpression of miR-98 targeted inhibiting platelet activating factor receptor-mediated microglial phagocytosis to attenuate neuronal death. Furthermore, we showed that neurons transferred miR-98 to microglia via EVs secretion after ischemic stroke, to prevent the stress-but-viable neurons from microglial phagocytosis. Therefore, we reveal that EVs derived miR-98 act as an intercellular signal mediating neurons and microglia communication during the brain remodeling after ischemic stroke. The present work provides a novel insight into the roles of EVs in the stroke pathogenesis and a new EVs-miRNAs-based therapeutic strategy for stroke.

## Introduction

Ischemic stroke, one of the most common disabling diseases, is mainly characterized by thromboembolic occlusion of the main artery supplying the brain. Clinically, rapidly recanalizing the occluded blood vessels and early re-establishing blood flow and tissue reperfusion could save part patients, who are eligible for thrombolysis and also expected to have the potential to improve functional outcome in the prognosis^1–3^. After stroke, the ischemic brain induces highly dynamic alterations, interacting processes and limited remodeling of the neurovascular unit, involving dysfunction of endothelial cells, neurons, glia, pericytes, and associated tissues proteins^4^. When neurons suffer threatens from ischemic stimuli, they not only activate self-preservation but also release extracellular “help me” signals to adjacent cells and induce them to shift into beneficial phenotypes, to limit damage^5^. Some findings indicate that endangered or damaged neurons can release a repertoire of signaling molecules to instruct surrounding cells including microglia, control microglial function, and regulate microglia-mediated phagocytosis and neuroprotection^6^. However, the signals involved in regulating the interactions between neurons and microglia remain unclear.

Currently, EVs grasp our eyes as a flexible medium in cell-to-cell interaction^7–9^. EVs, <150 nm in diameter, are secreted into the extracellular space and then fused to neighboring cells to release their contents^10,11^. Emerging evidence suggests that EVs play a significant role in intercellular communication by transferring protein and RNA cargo among components of the neurovascular unit after stroke^12^. Some researches indicate that EVs derived miRNAs play important roles in brain repair processes after stroke^13–15^. Surprisingly, the ratio of microRNAs (miRNAs) over total RNAs in EVs is higher than that in their parent cells^7,16^. MicroRNA-98 (miR-98), belonging to highly-conserved miRNAs, the let-7 group, has been reported to participate in the protection of blood–brain barrier function and the pathogenesis of ischemic stroke^17,18^. One study showed that only miR-98 was significant decreased in the cultured hippocampal neurons at 1 h post oxygen–glucose deprivation (OGD) 30 min, different from other miRNAs^19^, which indicated a specific role of miR-98 in stroke.

As is known to all, the brain is an “immune-privileged” organ, meaning that brain immunity is under tight control to protect nervous system from potential harmful immune reactions^5,20^. Microglia, the native immune cell in the central nervous system (CNS), once activated, exert key immune regulative function during ischemic stroke^21–23^. Accumulating evidence indicates that neurons could control microglial activation and influence microglial function, and then in turn microglia affect the neuronal survival during the ischemic stroke^1^. Microglia are the professional phagocytes in the brain and microglial phagocytosis is a double-edged sword^24^. Phagocytosis of dead neurons and neuronal debris is in part beneficial because it reduces inflammation^25^. However, they also engulf those dying or damaged salvageable neurons in the ischemic penumbra after ischemic stroke. Blocking the process of phagocytosis of stress-but-viable neurons is considered as a beneficial therapeutic strategy for ischemic stroke^26–31^.

Based on the above evidence, we hypothesize that neurons might release EVs containing miR-98, as a “help me” signal, to regulate microglial phagocytosis after ischemic stroke. The present work was to reveal the roles and the involved mechanisms of EVs derived miR-98 delivered from neurons to microglia in ischemic stroke.

## Results

### MiR-98 participates in the acute phase of ischemic stroke

**Fig. 1:**
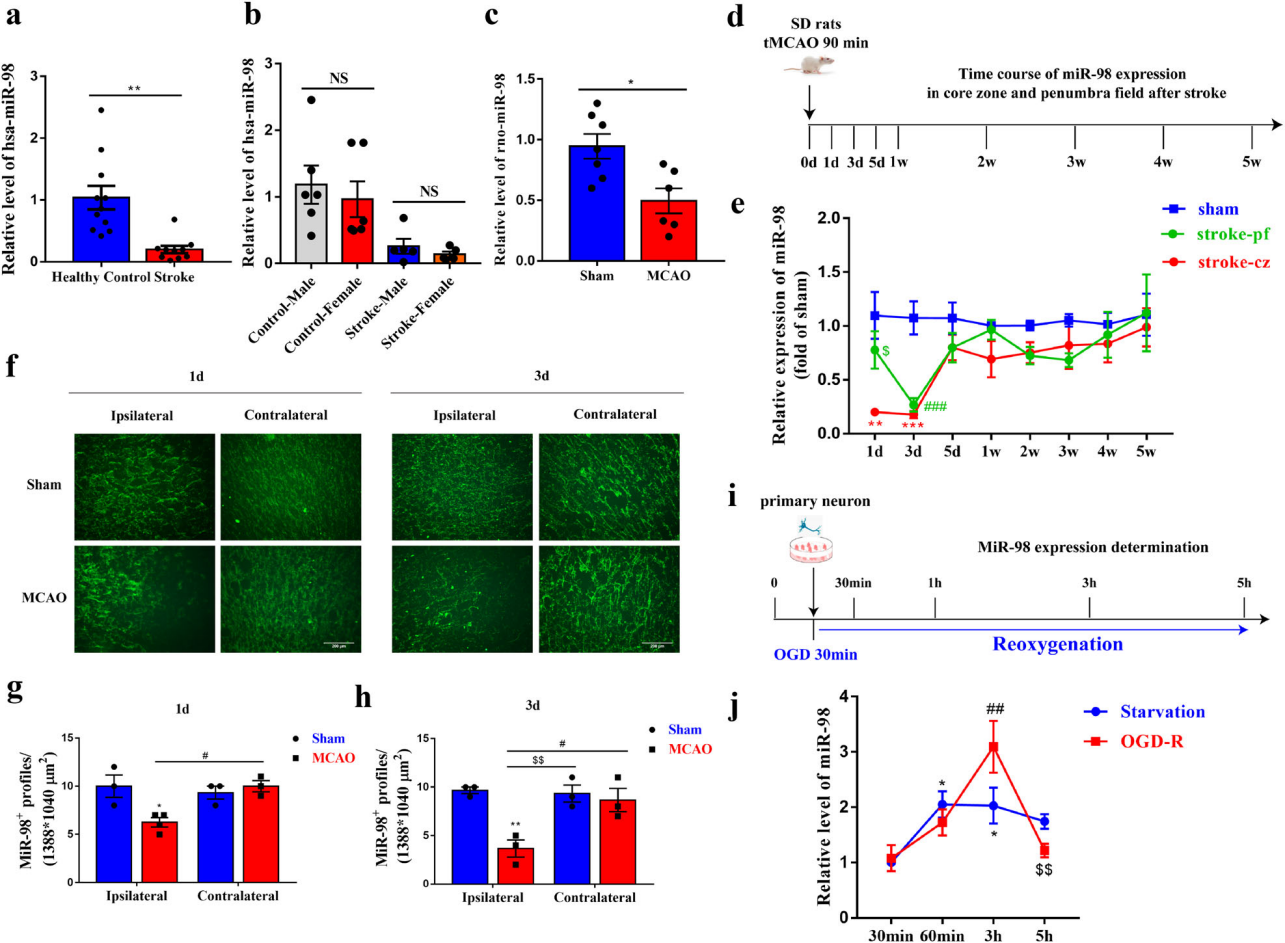
MiR-98 participates in the acute phase of ischemic stroke. **a**–**b** The expression of miR-98 in the serum of acute stroke patients (both male and female) vs. healthy controls. **c** The expression level of miR-98 in the serum of acute stroke rats vs. sham groups. **d**–**e** The expression level of miR-98 in the core zone and penumbra field of rats after ischemic stroke from 1 day to 5 week (*N* = 6 rats for each group). **f**–**h** Downregulation of miR-98 expression detected by fluorescence in situ hybridization (FISH) in ipsilateral regions at 1 day and 3 day after ischemic stroke compared with contralateral regions and sham group and quantification. Scale bar = 200 μm. (*N* = 3 rats for each group). **i**–**j** The determination of the miR-98 expression level alterations of primary neurons treated with 30-minute OGD and reoxygenation 30 min, 1 h, 3 h, and 5 h compared with starvation treatment (removing B27). (*n* = 3 independent experiments). Data are means±S.E.M., *n* = 8 mice/group. ^*^P, ^#^*P*, ^$^*P* < 0.05; ^**^P, ^##^*P*, ^$$^*P* < 0.01; and ^***^P, ^###^*P* < 0.001 vs. control.

First, in order to assess the clinical significance of miR-98 in ischemic stroke, we determined the expression level of miR-98 in serum from 10 randomly selected acute ischemic stroke patients enrolled in hospital and corresponding non-stroke healthy controls (Supplementary Table 1). We found that miR-98 expression level was obviously downregulated in the serum of acute ischemic stroke patients compared with healthy controls and there were no significant differences between males and females (Fig. 1a, b). In the ischemic stroke rat models induced by transient middle cerebral artery occlusion (tMCAO) (assessed by laser doppler flowmetry, Supplementary Fig. 1), the levels of miR-98 in the serum were also decreased compared with sham group (Fig. 1c). The above results indicated the potential role of miR-98 in the ischemic stroke.

To further determine the function of miR-98 in the critical period of ischemic stroke pathogenesis, we quantified temporal changes of miR-98 expression of ischemic stroke rats within 1 day to 5 weeks after stroke using RT-qPCR (Fig. 1d). The expression levels of miR-98 markedly decreased in the core zone of ischemic brain on the 1st day and 3rd day, and rose during the subsequent observation period. Notably, miR-98 expression maintained the same level as the sham groups in the penumbra field on the first day but dropped sharply on the third day after stroke (Fig. 1e). The same alterations were confirmed by staining results examined by fluorescence in situ hybridization (FISH) (Fig. 1f–h).

Next, we validated the findings in the primary cultured neurons from rat cortex treated with 30-min OGD and reoxygenation. We determined the early temporal alterations of miR-98 level at 30 minutes, 1 h, 3 h, 5 h after reoxygenation with the starvation treatment for control (Fig. 1i). Interestingly, we found that the expression level of neuronal miR-98 showed an increase and then dropped both in starvation groups and in OGD-treated groups. What’s more, we found miR-98 expression peaked at 3 h after reoxygenation and decreased markedly at 5 h after reoxygenation (Fig. 1j). These results suggest that miR-98 is involved in progression of ischemic stroke and might function as an endogenous protective factor in the early occurrence of ischemic stroke.

### MiR-98 can be packed into EVs transferred from neurons to microglia

**Fig. 2:**
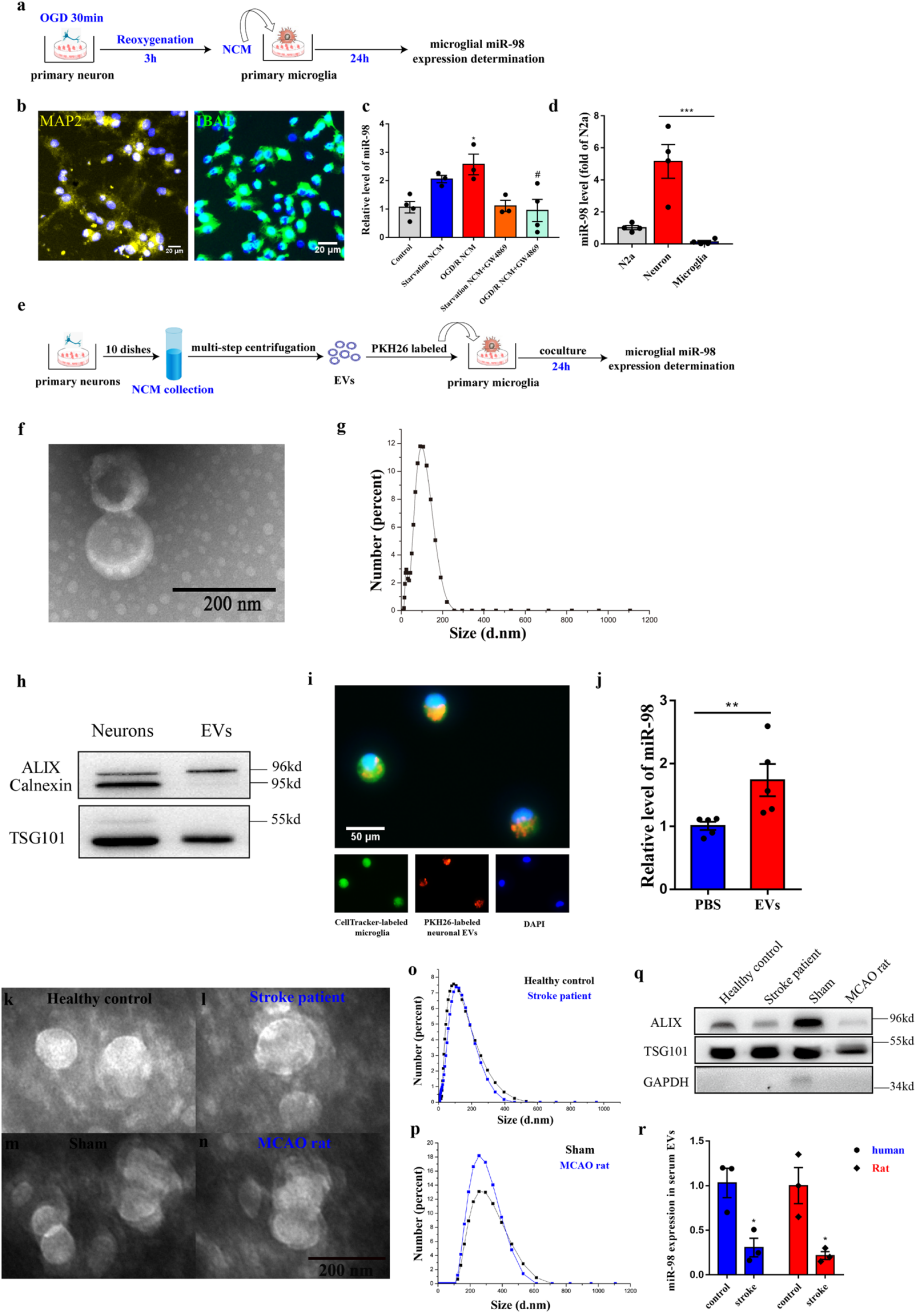
MiR-98 packed into EVs can be transferred from neurons to microglia. **a** The procedure of primary neuronal-conditional media (NCM) collection and coculture with primary microglia. **b** Positive rate of Rat primary cortical neuron (MAP2) and microglia (IBA1). **c** The relative miR-98 expression level of microglia after added with NCM from starvation and OGD-R treatment and GW4869 blocking. (*n* = 3 independent experiments). **d** The relative miR-98 expression level between neuron and microglia of purification culture, using the miR-98 expression of Neuro-2a (N2a) cells as a baseline. **e** The procedure of extraction of primary neuronal EVs and co-cultured with microglia. **f** Transmission electron micrograph (TEM) of neuronal EVs. **g** Size distribution of neuronal EVs based on Zetasizer Nano-ZS90 measurements. **h** Western blot for EVs markers Alix, TSG101, and Calnexin. **i** Representative images of internalization of PKH26-labeled neuronal EVs into the CellTracker^TM^ Green-labeled microglia 12 h after coculture. **j** Relative miR-98 level in microglia 24 h after incubated with PBS and neuronal EVs (extraction by ~80~100 ml neuronal media). (*n* = 5 independent experiments). Data are shown as means±S.E.M., ^*^P, ^#^*P* < 0.05; ^**^P < 0.01; ^***^P < 0.001 vs corresponding control. **k**–**n** Transmission electron micrograph (TEM) of serum EVs from both individuals and rats. **o**–**p** Size distribution of serum EVs from both individuals and rats. **q** Western blot for EVs markers Alix, TSG101, and GAPDH. **r** The relative miR-98 expression level between in serum EVs between both individuals and rats.

First, we collected primary neuronal-conditional media (NCM) from neurons (Fig. 2b, MAP2) which were treated with OGD 30 min and reoxygenation 3 h, when neuronal miR-98 expression peaked (Fig. 2a). The collected NCM were added into primary cultured microglia (Fig. 2b, IBA1) and miR-98 in microglia was examined after 24 h. We found miR-98 expression of microglia was significantly increased in microglia added NCM from neurons treated with OGD-R and has an increase trend (*p* = 0.177) in microglia added NCM from starvation treated neurons (Fig. 2c). We also demonstrated that miR-98 was expressed almost exclusively in neurons compared with microglia, using miR-98 expression of neuro-2a cells (N2a) as a baseline control (Fig. 2d). To verify the speculation that neuronal miR-98 could be packed into EVs and delivered from neurons to microglia, GW4869, a neutral sphingomyelinase inhibitor, was used to block EV secretion^32^. Interestingly, we found GW4869 (20 μM) could reverse the increase of miR-98 expression level in microglia after added NCM from neurons treated with starvation and OGD/R (Fig. 2c). These results suggested that neuronal miR-98 could be transferred via EVs secretion. Next, we isolated EVs from NCM and performed subsequent experiments (Fig. 2e). Transmission electron microscope (TEM) and Zetasizer Nano-ZS90 measurement identified the shape of “cup” with bilayer and size distribution of EVs (Zeta potential: −14.1 mv; Average size: 71.42 nm; polydispersity index: 0.410) (Fig. 2f, g). Western blotting analysis indicated that EVs markers ALIX and TSG101 were abundant in our extracted EVs without the endoplasmic reticulum marker Calnexin (Fig. 2h). Further, the yellow-orange fluorescent dye PKH26-labeled neuronal EVs were incubated with microglia labeled by CellTracker™ Green CMTPX Dye for 12 h. We observed PKH26-labeled neuronal EVs internalized into the microglia (Fig. 2i). Moreover, internalization of neuronal EVs significantly increased the miR-98 expression in microglia compared with phosphate-buffered solution (PBS) treated control groups (Fig. 2j) after 24 h incubation. Altogether, these results reveal that miR-98 could be transferred from neurons to microglia via EVs secretion. In addition, we measured the serum levels of EVs miR-98 in both individuals and rats, which were also characterized by TEM (Fig. 2k, l, m, m), size distribution (Fig. 2o, p), and western blotting (Fig. 2q). We found that the serum levels of EVs miR-98 were decreased in both individuals and rats likewise.

### In vivo transfer of miR-98 into microglia through neuron-secreted EVs after ischemic stroke

**Fig. 3:**
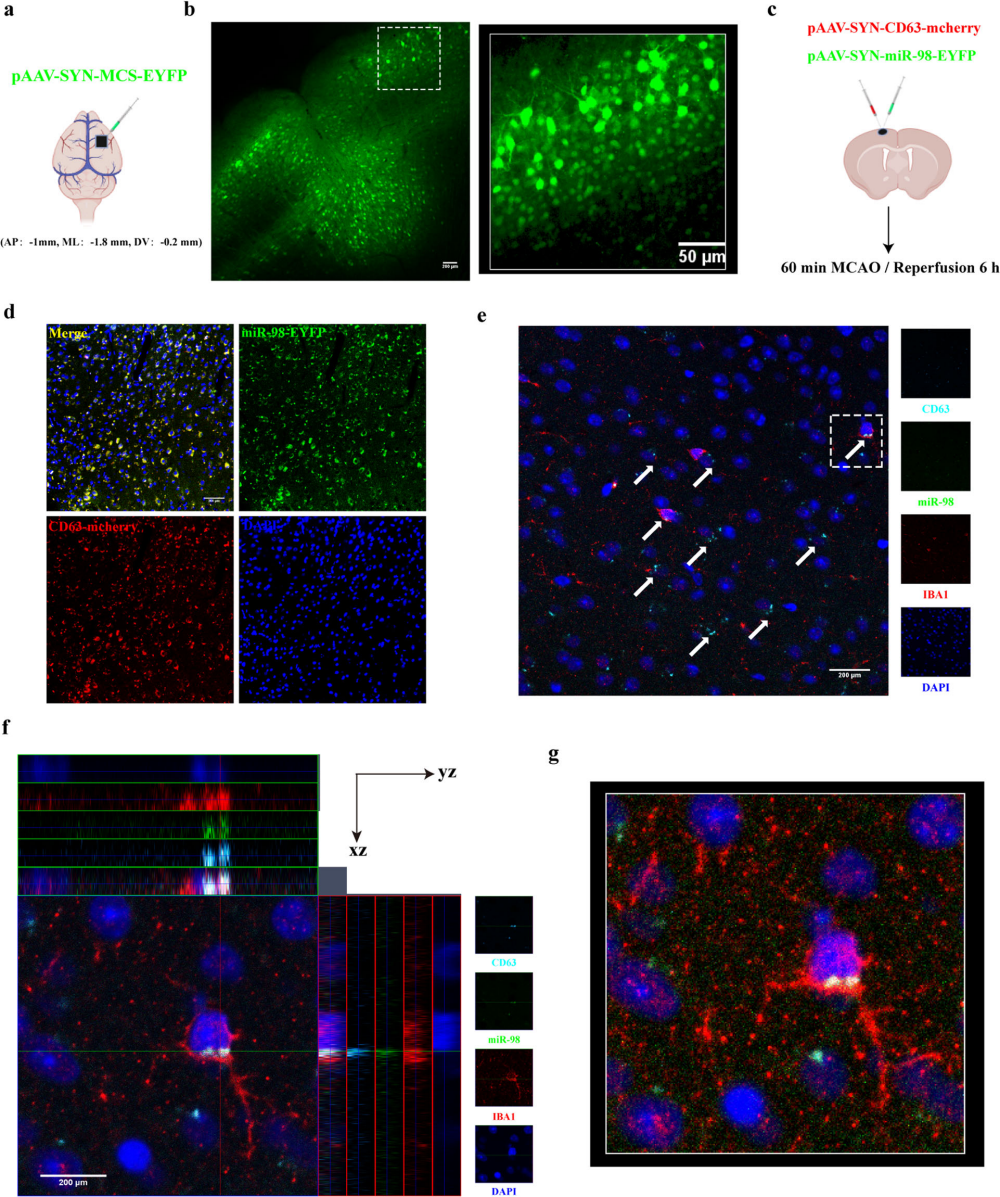
In situ illustration of neuronal EVs derived miR-98 internalized into microglia after ischemic stroke. **a** The injection of control virus pAAV-SYN-MCS-EYFP-3FLAG (9.46 × 10^12^, 0.5 μL) into cortex of P20 C57BL/6 J mice (AP:-1mm, ML:-1.8 mm, DV:-0.2 mm). **b** The identification of the region and efficiency of virus infection (left, Scale bar = 200 μm) and the 3D reconstructions of Z-stack images in white dotted box acquired by a two-photon Microscope (right, 100 μm thickness). **c** pAAV-SYN-CD63-mCherry-3FLAG (1.81 × 10^13^, 0.3 μL) and pAAV-SYN-EYFP-3FLAG-miR-98 (8.36 × 10^12^, 0.3 μL) were simultaneously injected into the same coordinates of control virus and subsequent tMCAO model 3 weeks later. **d** The representative confocal images of CD63-mCherry co-labeled with miR-98-EYFP and DAPI on paraffin slices (5 μm thickness, Scale bar = 200 μm). **e**–**g** The representative confocal images, amplification in white dotted box and colocalization analysis, and 3D reconstructions of CD63-mCherry co-labeled with miR-98-EYFP and IBA1 on paraffin slices 6 h after ischemic stroke acquired by a confocal microscopy. (5 μm thickness, Scale bar = 200 μm) (*n* = 3 independent experiments). The white arrows represent the CD63-mCherry and miR-98-EYFP were co-labeled.

To further confirm whether neurons could transfer miR-98 to microglia via EVs secretion, the mature neuron-targeted adeno-associated virus (AAV, SYN promoter, AAV2/9) was used to label neuronal EVs. A previous study reported that CD63 could be applied to label EVs^33^. Based on this, we injected the viruses pAAV-SYN-CD63-mCherry-3FLAG combined with pAAV-SYN-EYFP-3FLAG-miR-98 into the cortex of p20 C57BL/6 J mice (Fig. 3c), to label the neuronal EVs and miR-98, respectively. pAAV-SYN-MCS-EYFP-3FLAG was used as control label (Fig. 3a). We identified the infection region and efficiency of virus injection with immunofluorescence staining and the 3D reconstructions of Z-stack images (Fig. 3b). The colocalization of CD63-mCherry and miR-98-EYFP showed that these neurons expressed miR-98 (Fig. 3d). Three weeks after virus injection, the mice were exposed to tMCAO to establish the ischemic stroke model. After reperfusion 6 h, we found that CD63-labeled and miR-98-EYFP-labeled neuronal EVs appeared outside of the neurons and internalized into IBA1-labeled microglia in the ipsilateral regions obviously (Fig. 3e). Very clearly, we observed the colocalization of CD63-labeled neuronal EVs, neuronal miR-98 and microglia (Fig. 3f, g) with Z-stack images and 3D reconstructions of the white dotted regions acquired by a two-photon microscope, which could be observed 3 h after reperfusion (Supplementary 3a–c). Therefore, we confirm that neurons can transfer miR-98 to microglia via EVs secretion after ischemic stroke.

### Overexpression of miR-98 can prevent stressed but salvageable neurons from microglial phagocytosis targeted inhibiting PAFR after stroke

**Fig. 4:**
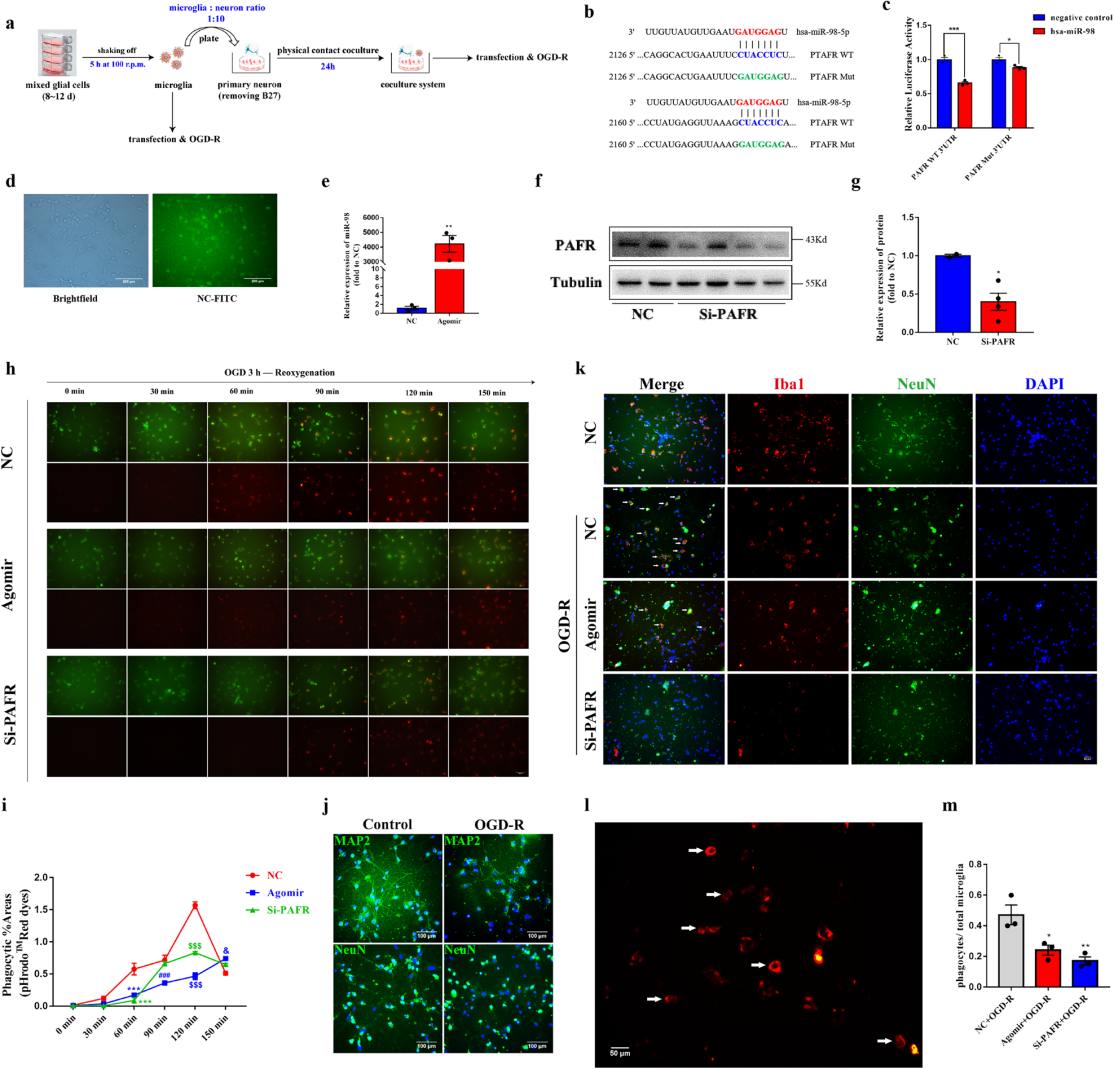
MiR-98 could inhibit the microglial phagocytosis of neuron after OGD/R in vitro. **a** The procedure of primary microglia transfection, OGD/R and physical contact co-cultured with neuron. **b** The target sequence of hsa-miR-98 and WT & Mut PAFR 3′-UTR. **c** The effect of hsa-miR-98 on PAFR expression via dual-luciferase report system. **d** The transfection efficiency of negative control (NC-FITC). **e** The transfection efficiency of miR-98 agomir. **f**–**g** The transfection efficiency of PAFR-siRNA. **h**–**i** The CellTracker^TM^ Green-labeled microglial phagocytosis of pHrodo^TM^ Red Dextran dye after treated with OGD 3 h and reoxygenation during the time course from 30 min to 150 min and quantification of the phagocytic %area. (Scale Bar = 100 μm). **j** Representative immunological staining of NeuN and MAP2 after primary neuron treated with 2 h OGD and reoxygenation for 24 h. (Scale Bar = 100 μm). **k** The representative images of the effects of overexpressing miR-98 and downregulating PAFR on the microglial phagocytosis of neurons after treated with OGD 2 h and reoxygenation for 24 h. The white arrows mean representative microglial phagocytosis of neurons. (Scale Bar = 50 μm). **l**–**m** The representative phagocytes of microglia and quantification of the percentage of phagocytes/total microglia numbers. (Scale Bar = 50 μm) Data are shown as means±S.E.M., ^*^P, ^&^*P* < 0.05; ^**^P < 0.01; ^***^P, ^###^*P*, ^$$$^*P* < 0.001 vs corresponding control. (*n* = 3 independent experiments).

To reveal the impacts that neurons transferred EVs derived miR-98 to microglia after ischemic stroke, we first study the roles of miR-98 in the primary cultured microglia (Fig. 4a). Targetscan software and miRbase database were used to predict that platelet activating factor receptor (PAFR) was one of the target genes of miR-98. Transcriptional profiling analysis in a database of cell type expression categories in the cerebral cortex of mice established by Zhang Y et al.^34^ showed that PAFR was expressed almost exclusively in microglia compared with neurons in the brain. We identified that miR-98 could target and regulate PAFR through binding with the 3′-UTR of PAFR using dual-luciferase reporter gene assay (Fig. 4b, c). Several studies indicated that PAFR is involved in the process of efferocytosis (phagocytosis of apoptotic cells) and engaged in microglial phagocytosis of synapses in the multiple sclerosis^35,36^. However, the role of microglial PAFR in ischemic stroke remains unclear. Therefore, we overexpressed miR-98 and downregulated PAFR, respectively (Fig. 4d–g) to identify the effects of miR-98 targeted regulating PAFR on the microglia phagocytosis.

The pH-sensitive rhodamine-based pHrodo™ Red dyes, used as specific sensors of endocytic and phagocytic events, will undergo a marked increase in fluorescence in response to an environmental shift from extracellular high pH to intracellular low pH. After 48 h transfection of miR-98 or PAFR, the pHrodo™ Red dyes were added into CellTracker^TM^ Green-labeled microglia before OGD/R treatment. The treated microglia were examined every 30 min by a fluorescence microscope. The time course of increased red fluorescence documented the accumulation of pHrodo-conjugated zymosan bioparticles. We found microglial phagocytosis peaked at 120 min after OGD 3 h and reoxygenation. Both miR-98 overexpression and PAFR downregulation could inhibit the red bioparticles phagocytosis of microglia and the increases of red fluorescence (Fig. 4h, i). Finally, we investigated whether miR-98 overexpression could prevent neurons from microglial phagocytosis. As shown in Fig. 4j and k, microglia were “friendly” symbiotic with surviving neurons before OGD/R treatment and microglia shifted into phagocytes (Fig. 4l) and attacked injured neurons after OGD-R treatment. MiR-98 overexpression or PAFR downregulation could inhibit the microglial phagocytosis of surviving neurons (Fig. 4k, m). The results suggest that miR-98 targeting PAFR could prevent salvageable neurons from microglial phagocytosis after OGD/R treatment.

**Fig. 5:**
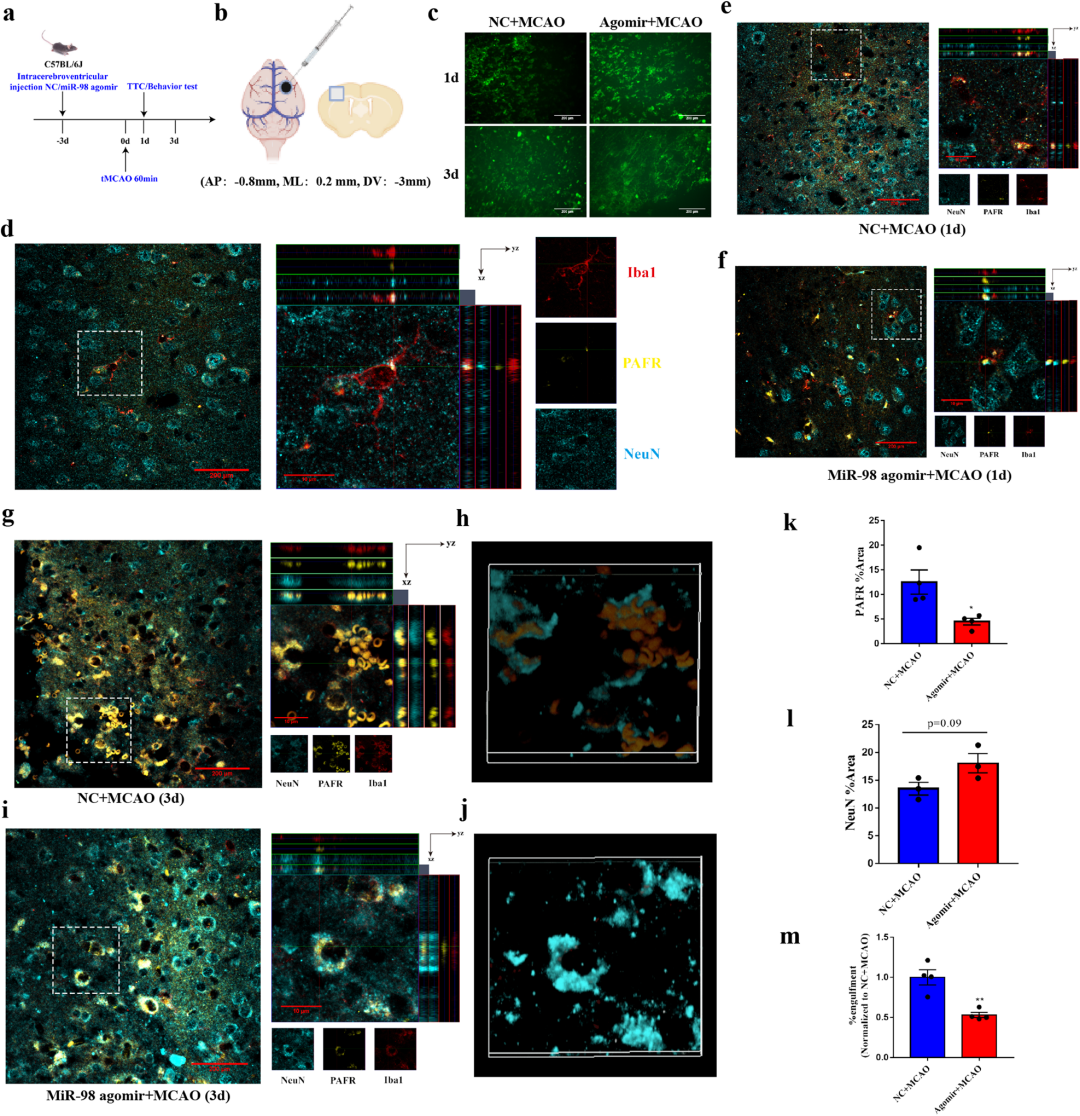
MiR-98 can inhibit the microglial phagocytosis of “stress but viable” neurons after ischemic stroke through downregulating PAFR. **a**–**b** The procedure and localization of intracerebroventricular injection of miR-98 agomir (negative control) in mice. **c** The representative of miR-98 expression in the ipsilateral regions 1 day and 3 days after ischemic stroke with miR-98 agomir treatment in FISH. (Scale Bar = 200 μm). **d** The representative immunostainings and Z-stack images of NeuN, IBA1, and PAFR colocalization in mice brain slices of sham group. **e**–**f** The representative immunostainings and Z-stack images of NeuN, IBA1, and PAFR colocalization in mice brain slices of mice with NC/miR-98 agomir treatment 1 day after ischemic stroke. **g**–**j** The representative immunostainings and Z-stack images, 3D reconstructions of NeuN, IBA1, and PAFR colocalization in mice brain slices of mice with NC/miR-98 agomir treatment 3 days after ischemic stroke. These Z-stack images and 3D reconstructions of original pictures were required by a Confocal Microscopy or two-photon Microscope. (*n* = 3 independent experiments). **k**–**m** Quantification of the fluorescent %area of PAFR and NeuN and microglial engulfment 3 days after ischemic stroke with NC/miR-98 agomir treatment. Data are shown as means±S.E.M., ^*^P < 0.05 vs corresponding control.

To further address our speculations in vivo, miR-98 agomir was injected into the lateral ventricle 3 days before C57BL/6 J mice were subjected to focal cerebral ischemia (Fig. 5a, b). Previous studies indicated that phagocytic microglia were observed soon 1 day after MCAO, peaked at 3 days after MCAO^37^. Blocking microglia engulfing stress but viable neurons may be beneficial after ischemic stroke^28,30^. Therefore, we tested microglial phagocytosis on the first day and 3rd day after ischemic stroke. FISH was used to determine the expression of miR-98 (Fig. 5c). NeuN and IBA1were utilized to label neurons and microglia. The results showed that in normal conditions, microglia were branched and some were adjoined with neurons, with a rather low expression of PAFR (Fig. 5d). On the first day after ischemic stroke, the microglia activated and could phagocytize some dead neuronal fragments. At this time, miR-98 agomir treatment did not obviously affected microglial phagocytosis in the ipsilateral lesions (Fig. 5e, f). On the third day after ischemic stroke, microglia abundantly shifted into phagocytes and vigorously ate stressed but viable neurons in the lesions. Strikingly, Z-stack images and 3D reconstructions showed that miR-98 agomir treatment significantly inhibited microglial phagocytosis of dying neurons in the ipsilateral lesions (Fig. 5g–j, m, Supplementary Fig. 5 & 8, Supplementary video 1, 2). Collectively, the above results in vitro and in vivo suggest that overexpression of miR-98 blocks microglial phagocytosis of stress but salvageable neurons possibly by inhibiting PAFR (Fig. 5k) and subsequently attenuate neuronal death (Fig. 5l) in acute ischemic stroke.

### Overexpression of miR-98 can reduce neuronal damages in ischemic stroke in in vitro and in vivo

**Fig. 6:**
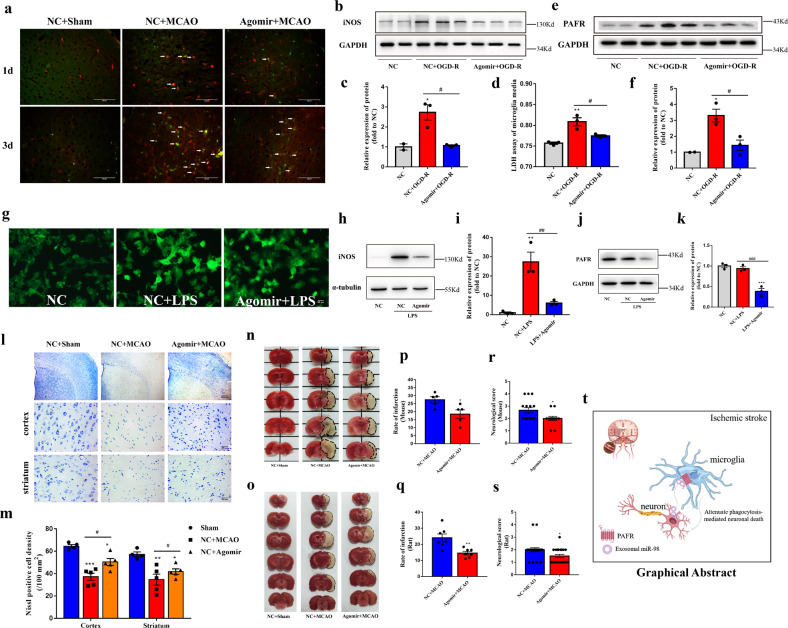
Overexpression of miR-98 can reduce neuronal damages in ischemic stroke in in vtro and in vivo. **a** The representative immunostaining of iNOS (green) and IBA1 (red) of mice brain slices 1d and 3d after ischemic stroke. The white arrows are iNOS and IBA1 colocalization. (scale bar = 200 μm). **b**–**c** The protein expression level of iNOS in microglia after OGD-R treatment and miR-98 agomir transfection, and quantification. **d** The lactate dehydrogenase (LDH) assay of microglia after OGD-R. **e**–**f** The protein expression level of PAFR in microglia after OGD-R treatment and miR-98 agomir transfection, and quantification. **g** The representative immunostainings (IBA1) of morphology of microglia. (Scale Bar = 50 μm). **h–k** The protein expression level of iNOS, PAFR and quantification. (*n* = 3 independent experiments). **l**–**m** The Nissl staining of cells of ipsilateral cortex and striatum and quantification. (*n* = 5 independent experiments). **n**–**s** The representative TTC stainings of the mice and rats brain sections and quantification of TTC stainings (*N* = 6 mice for each group, **f** rats for each group) and neurological function evaluations (*N* = 14 mice for each group, 20 rats for each group). **t** The schematic of neuronal EVs miR-98 transfer hypothesis between neurons and microglia after ischemic stroke. Data are shown as means±S.E.M., *P, ^#^*P* < 0.05; **P, ^##^*P* < 0.01; ***P < 0.001 vs corresponding control.

Microglia activation can cause neurotoxicity via excess nitric oxide (NO) generation by inducible NO synthase (iNOS). High levels of NO induce neuronal death by causing inhibition of mitochondrial cytochrome oxidase in neurons^38^. Accordingly, we detected the iNOS expression of microglia after ischemic stroke. The results showed that miR-98 agomir inhibited iNOS expression significantly on the 3rd day after ischemic stroke (Fig. 6a). Simultaneously, we verified that miR-98 agomir reduced the expressions of iNOS in microglia (Fig. 6b, c) and relieved OGD-R-induced cell injury determined by lactate dehydrogenase assay (Fig. 6d). Previous studies reported that PAFR-mediated signaling pathway regulated the iNOS production^39^, our results also demonstrated that miR-98 agomir downregulated PAFR and reduced the expression of iNOS (Fig. 6e, f). In addition, we observed the effects of miR-98 in the LPS-induced inflammatory model of microglia. We found overexpression of miR-98 inhibited LPS-induced microglial activation (Fig. 6g). Overexpression of miR-98 could downregulate PAFR and thereby reduce the expression of iNOS (Fig. 6h–k), whereas could not reverse the expression of CD206 which is thought as an anti-inflammatory marker (Supplementary Fig. 6). Altogether, these results indicated that miR-98 can reduce iNOS production by inhibiting PAFR and attenuate inflammatory damage.

We further demonstrated that miR-98 could alleviate neural injury both in mice and rats after ischemic stroke. The results showed that miR-98 agomir could significantly increase the Nissl positive cells, suggesting relieving the neuronal damages induced by ischemia both in cortex and striatum (Fig. 6l, m). Subsequently, miR-98 agomir reduced the infarctions and attenuated the neurological deficits both in mice and rats (Fig. 6n–s).

## Discussion

We show herein that neurons communicate with microglia via EVs to remodel the brain after ischemic stroke. Our results provide evidences that EVs derived miR-98 can act as an intercellular signal to regulate microglia engulfing neurons after ischemic stroke (Fig. 6t). Our data support the concept that non-cell autonomous signaling is vital for CNS recovery after injury or disease.

Although the pathogenesis of ischemic stroke has been investigated for decades, preventing delayed neuronal death is still the therapeutic direction. However, there are currently no precise blood-based biomarkers with clinical utility for acute ischemic stroke. Studies reported that miRNAs showed promise for ischemic stroke owing to their cell type-specific expression patterns and stability in peripheral blood^40,41^. Therefore, we attempted to explore their utility as an early diagnostic marker and potential therapeutic value for ischemic stroke. In our study, we first found miR-98 was decreased obviously in the serum of acute ischemic stroke patients. The similar decrease of miR-98 expression was also observed in the serum of ischemic stroke rats induce by tMCAO. Furthermore, we demonstrated that the serum levels of EVs miR-98 were decreased in both individuals and rats likewise, which indicate that miR-98 might be a potential biomarker for ischemic stroke.

MiR-98, as a member of let-7 group, has been reported to participated in several neurological diseases such as Alzheimer’s Disease^42^, neuroblastoma^43^ and neuro-inflammatory conditions^17^. Whereas, the specific effects and mechanisms of miR-98 involved in the pathophysiological progress of ischemic stroke remain unknown. To determine the critical periods and function of miR-98 participating in the ischemic stroke, we utilized rats to establish ischemic stroke model and observed the changes of miR-98 in the penumbra field of rats 1 day to five weeks after ischemia. Of note, we observed that the expression level of miR-98 kept from decrease on the 1st day but dropped significantly on the 3rd day post-ischemia. Simultaneously, we found miR-98 expression peaked at 3 h and dropped markedly at 5 h after OGD/R. These data suggest that miR-98 could serve as an endogenous protective factor for the acute phase of ischemic stroke. Previous studies mentioned that ischemic neurons could not only activate self-preservation but also release extracellular “help me” signals to adjacent cells to limit damage. Based on this, we guessed that miR-98 could function as a signal mediating communication between neurons and other neural cells after ischemic stroke. EVs are a novel form of information exchange among neural cells within the nervous system, via carrying and transferring cargo miRNAs^44–47^, and regulate brain remodeling process after ischemic stroke^12,48^. Our results showed that the expression alteration of miR-98 was consistent to the time of microglial phagocytosis change^37^, suggesting that miR-98 might be involved in the neurons and microglia interaction. Microglia are brain-resident immune cells that mediate key functions to support the CNS. Microglia express a wide range of molecular sensors, which recognize exogenous or endogenous insults and initiate an immune response^21,49^. In addition, microglia also act as guardians in the brain by promoting phagocytic clearance and providing trophic support to ensure tissue repair and maintain cerebral homeostasis^50^. Microglial activation is considered as an early, sensitive and credible signal for neuronal damage in progress of ischemic stroke^37^. Therefore, we hypothesized that neuronal EVs derived miR-98 could act as a “help me” signal and be released to microglia.

Our present work demonstrated that neurons could transfer miR-98 via EVs secretion to prevent the stress-but-viable neurons from microglial phagocytosis after ischemic stroke. PAFR was reported to regulate phagocytosis in the neurological diseases as mentioned previously^35,36^. In our work, we performed experiments in vitro and in vivo to demonstrated that miR-98 could target PFAR and thereby inhibit the microglial phagocytosis, and thereby attenuate ischemia/OGD-induced neuronal death. Finally, we confirmed that miR-98 agomir given ahead could alleviate neurological deficit and infarction of ischemic stroke in rats and mice, which further confirmed that miR-98 exerted endogenous protective effects. Therefore, our data show that miR-98 regulates microglial phagocytosis through targeting PFAR, and thereby plays neuroprotection in stroke.

Taken together, we reveal that EVs derived miR-98 acts as an intercellular signal mediating neurons and microglia communication during the brain remodeling after ischemic stroke. The present work provides a novel insight into the roles of neuronal EVs in the stroke pathogenesis and a new EVs derived miRNAs-based therapeutic strategy for stroke. Moreover, the serum level of miR-98 might be a potential indicator for stroke diagnosis and prognosis.

## Materials and methods

### Animals

All experiments and protocols were approved by Institutional Animal Care and Use Committee (IACUC) of Nanjing Medical University and in accordance to the rules of the Experimental Animal Application Criteria. The approval no. IACUC-1912041. Sprague-Dawley (SD) male rats weighing 250 ± 10 g and 6–8-week-old C57BL/6 J male mice weighing 25 ± 2 g were purchased from the Animal Resource Center of the Faculty of Medicine of Nanjing Medical University for use in the experiment. All the animals were kept in a standardized environment (temperature 25 ± 2 °C, humidity 55 ± 10%), maintained on a 12 h light/dark cycle (lights on from 20:00 to 08:00) and housed with access to food and water ad libitum. All the experimental animals were allowed to adapt to the new environment for 1 week and fasted for 12 h before the experiments. All the animals were allocated to experimental groups and processed with no randomization and the investigator was blinded to the group allocation.

### Human samples

The serum samples of acute stroke patients were provided by Department of Neurology and age-matched healthy control provided by health examine center, the Second Affiliated Hospital of Nanjing Medical University. The information of human subjects is provided in Supplementary Table S1. All the subjects were informed consent for the sample collection. The study was approved by the Medical Ethical Committee of Nanjing Medical University (no. 2017-032). Equal volumes of serum from stroke patients and healthy control (200 μL) were loaded for real-time quantitative reverse transcription–polymerase chain reaction (qRT-PCR) detections.

### Quantitative reverse transcription–polymerase chain reaction

Real-time qRT-PCR was carried out using the SYBR Green mixture (TaKaRa, Japan) in a QuantStudio 5 system (Thermo Fisher Scientific, USA). The Total RNA was extracted from serum, tissues and cells by using a TRIzol reagent (Life Techonologies, 15596018) and prepared for quantitative reverse transcriptase PCR by using MasterMix (TaKaRa, Japan). Reverse transcription was performed using the All-in-One^TM^ miRNA First-Strand cDNA Synthesis Kit (GeneCopoeia, Cat. No: QP013). Total RNA (800 ng) was reverse-transcribed with 4 μL 5 × PAP/RT buffer, 0.8 μL RTase Mix, 0.8 μL 2.5 U/μL Poly A Polymerase and RNasefree water added to 20 μL. A reverse transcription reaction was performed in a thermal cycler as follows: 37 °C for 60 min, 85 °C for 5 min, and held at 4 °C forever. The cycling conditions were as follows: denaturation at 95 °C for 30 s, followed by 40 cycles of DNA synthesis at 95 °C for 5 s and 60 °C for 34 s. U68 was used as an endogenous control, and the relative expression of target genes was determined using the 2^−ΔΔct^ method. The All-in-One^TM^ miRNA Universal Adaptor PCR Primer (GeneCopoeia, Cat NO: P01011A) was used as the reverse primer sequence of U68 and miR-98. The forward primer sequence of rno/hsa-U68 used the All-in-One^TM^ miRNA qPCR Primer (GeneCopoeia, Cat NO: RmiRQP9013; HmiRQP9071) and the forward primer sequence of rno/hsa-miR-98 used the All-in-One^TM^ miRNA qPCR Primer (GeneCopoeia, Cat NO: RmiRQP0853; HmiRQP9021). The serum EVs-miRNAs detections were normalized to spiked cel-miR-39 as described previously^51^.

### Focal cerebral ischemia/reperfusion model

SD male rats and C57BL/6 J male mice were both used for focal cerebral ischemia/reperfusion experiments. We used a tMCAO model by means of an intraluminal suture. In brief, animals were anesthetized with isoflurane and the right external carotid artery was exposed and a nylon monofilament coated with silicon resin (Doccol) was introduced through a small incision and advanced to the carotid bifurcation along the internal carotid artery into the middle cerebral artery. The monofilament was withdrawn to restore blood flow to the middle cerebral artery territory 1 h (mice)/1.5 h (rats) after the occlusion. Focal cerebral ischemia was assessed by Laser Doppler flowmetry^52^. Neurological function was evaluated using a four-point scale neurological score method (0, no observable deficit; 1, forelimb flexion; 2, decreased resistance to lateral push and immediate circling; 3, severe rotation progressing into loss of walking or righting reflex; 4, not walking spontaneously and some degree of consciousness)^53^. Animals dead in the model were excluded. The identification of ischemic core zones and penumbra field are performed as previously described^54^.

### Fluorescence in situ hybridization

Animals were anesthetized with isoflurane and perfused with PBS followed by 4% paraformaldehyde (PFA). After post fixation with 4% PFA overnight, the brains were dehydrated using a graded series of alcohol, cleared in xylene, and embedded in paraffin. Then, coronal slices (5 µm) were cut by a rotary microtome (LEICA, RM2245, Heidelberg, Germany). The following steps followed the BersinBio^TM^ Fluorescence in Situ Hybridization Kit Instruction Manual. In brief, Xylene processing sample for 15 minutes at room temperature (every 5 minutes change the xylene). Incubate twice with 100% ethanol for 5 minutes. Then dry the sample. Then add proteinase K mixture (0.2 μL proteinase K, 20 μL Tris-Hcl, pH 7.6, 80 μL RNasefree water) on sample and incubate 10–15 minutes at 37°C. Rinse slides twice with PBS for 5 minutes at room temperature. Incubate sample for 5–8 minutes with preheating denaturing solution (70% formamide in 2× SSC [17.6gNacl,8.8 g sodium citrate in 1 L sterilized water]) at 78 °C. Incubate with 70% Ethanol for 5 min, 85% Ethanol for 5 minutes, and 100% Ethanol for 5 minutes at −20 °C. Then dry the sample. Add 2 μl miR-98-5p FITC Probe (BersinBio^TM^, QD355) to the remaining 18 μL Probe Diluent (60% deionized formamide, 500 μg/mL yeast tRNA [Invitrogen, 15401011], 50 μg/mL Heparin sodium salt [Aladdin, 9041-08-1], 0.1% Tween-20, 20× SSC, 9.2 mM Citric acid solution, pH 6.0) to obtain 20 μL Hybridization reaction mixture. Mix well and denatured at 78 °C for 5 minutes. Then incubate for 5 minutes at 37 °C. Then add 10–15 μl Hybridization reaction mixture on sample. Add lid and incubate for 16 h to 20 h at 37 °C. Rinse slides twice with preheating washing buffer for 5 minutes at 53 °C. Rinse slides twice with preheating 2× SSC for 5 minutes at 37 °C; 0.5×SSC for 15 minutes at 37 °C; 0.2× SSC for 15 minutes at 37 °C; Rinse slides with PBS for 5 minutes at room temperature. Samples can be analyzed in a drop of fluorescence decay mounting medium (Beyotime Biotechnology, P0126) under a fluorescence microscope.

### Primary neuron cultures

Primary neuron cultures were prepared from cerebral cortices of embryonic day (E) 16~18 Sprague-Dawley rat embryos^44^. In brief, embryos were removed from maternal rat anesthetized with isoflurane and euthanized by decapitation. Cerebral cortices were dissected and digested with 0.125% trypsin (Life Technologies, 27250-018) and 0.025% DNase I (Biofroxx, 1121MG010) at 37 °C for 15 min. Dissociated cells were filtered with 80-μm cell strainer, collected by centrifugation (1000 rpm for 5 min) and plated on dishes (Corning, 430167) or 24-well plates (Corning, 3524) coated with 0.01 mg/mL Poly-d-lysine (Sigma, P6407) and cultured in Dulbecco’s Modified Eagle Medium (DMEM, Life Technologies, 11965-084) containing 10% fetal bovine serum (FBS, Life Technologies, Australia, 10099-141), 50 U/mL penicillin–streptomycin (Life Technologies, 15140122). At 6 h after seeding, the medium was changed to Neurobasal medium (Life Technologies, 21103-049) supplemented with 2% B27 (Life Technologies, 17504044) and 50 U/mL penicillin–streptomycin. Neurons were maintained at 37 °C in a humidified incubator of 95% air and 5% CO_2_ and grown for 10~12 days with half of the media replaced every 2 days. The purity of neuron cultures was determined by immunocytochemical staining using an antibody against microtubule‐associated protein‐2 (MAP‐2), >90%.

### Primary microglia cultures and transfection

Primary microglia cultures were isolated from 1-day-old postnatal SD rats. In brief, primary cultures of glial cells were obtained from the cerebral cortices, which were earlier digested by 0.125% trypsin at 37 °C for 15 min and seeded into poly-d-lysine-coated 25-cm^2^ culture flasks (Corning, 430639). The cultures were maintained for 10 days in DMEM supplemented with 10% FBS and 50 U/mL penicillin–streptomycin at 37 °C in a humidified incubator with 5% CO_2_ air atmosphere. Media were replaced every 2~3 days and grown for 9~10 days. Microglia were separated from the mixed primary culture by shaking off for 5 h at 100 rpm and then planked in plates with DMEM medium containing 10% FBS and 50 U/mL penicillin–streptomycin. Before the experiments, the percentage of the primary microglia was confirmed by Ionized calcium binding adapter molecule 1 (Iba1, WAKO, 019-19741) staining with over 97% purity.

Primary microglia were transfected in a 6/24-well plate with miRNA agomir, miRNA antagomir and siRNAs purchased from GenePharma (Shanghai, China) using siRNA-Mate (GenePharma, G04002), according to the manufacturer’s instructions. The sequences are as follows.

Rno/mmu-miR-98 agomir:

Forward: 5′-UGAGGUAGUAAGUUGUAUUGUU-3′

Reverse: 5′-CAAUACAACUUACUACCUCAUU-3′

Rno/mmu-miR-98 antagomir:

5′-AACAAUACAACUUACUACCUCA-3′

Rno-Ptafr siRNA:

Forward: 5′-CCAACUUCCAUCAGGCUAUTT-3′

Reverse: 5′-AUAGCCUGAUGGAAGUUGGTT-3′

Negative control-FITC:

Forward: 5′-UUCUCCGAACGUGUCACGUTT-3′

Reverse: 5′-ACGUGACACGUUCGGAGAATT-3′.

### Oxygen and glucose deprivation and reoxygenation (OGD-R)

OGD experiments were performed using an incubator (Thermo Fisher Scientific, Waltham, USA) with premixed gas (95% N_2_ and 5% CO_2_) kept at 37 °C. To initiate OGD, cells culture medium was replaced with deoxygenated, glucose-free DMEM (Life Technologies, 11966-025). After OGD, the cells were then transferred to a normoxic incubator (95% air and 5% CO_2_) replaced with normal medium. Although the time of OGD challenge and reoxygenation could be adapted according to different cells and culture system.

### NCM preparation

NCM was harvested from neurons grown for 10~12 days replaced with Neurobasal medium without B27 supplement (starvation control) or OGD 30 min and reoxygenation 3 h plus GW4849 (EV secretion blocker, 20 μM, Santa Cruz Biotechnology, sc-218578). NCM from neurons was cleared by centrifugation (1000 rpm for 5 min) and then diluted to 3:1 by mixing with microglial complete medium to treat microglia. NCM was collected simultaneously and used in parallel without storage. After treated 24 h, microglia were collected for the next detections.

### Primary neuron-microglia physical coculture

To generate neuron-microglia physical coculture^55^, cortical neurons (1 × 10^6^ cells/mL) were cultured in 24-well plates and grown for 8~9 days replaced with neurobasal medium without B27 supplement, which inhibits the growth of glial cells. Primary microglia (1 × 10^5^ cells/mL) were seeded and cultures 1:10 with neurons. After 24 h, the coculture system was subjected to OGD/R treatment.

### Isolation of serum and neuronal EVs

Neuronal EVs were isolated from the harvested conditioned medium of rat cortical neurons cultured 10~12 days in vitro. Every 5–6 × 10^6^ neurons were cultured in a 100-mm dish and 10 mL NCM were collected. The EVs were harvested from 10 dishes NCM supernatant (100 mL) and isolated by multi-step centrifugation as previously reported with minor modification. Serum EVs were isolated as the same. Briefly, the supernatant was centrifuged at 300 × *g* for 10 min, 2000 × *g* for 10 min, and 16,500 × *g* for 30 min at 4 °C to remove cells and debris and then filtered using a 0.22-μm filter (Millipore, SLGV033RB). The filtrate was centrifuged at 140,000 × *g* for 2 h at 4 °C in a Type Ti70 rotor using an L-100XP ultracentrifuge (Beckman Coulter, Brea, CA, USA). The EV pellet was resuspended in PBS and ultracentrifuged again at 140,000 × *g* for 2 h. The isolated pellets were suspended in sterile 1× PBS for EV characterization and culture experiments^56^.

### Identification of EVs by TEM, nanoparticle tracking analysis (NTA), and western blotting

The EVs were diluted in sterile PBS, fixed with 1% glutaraldehyde, applied onto a carbon-coated copper grid, and stained with 1% phosphotungstic acid. A TEM (Tecnai Spirit Biotwin) was employed to observe the specimens. The average particle size and zeta potential of neuronal and serum EVs were investigated with a Zetasizer (Nano-ZS90, Malvern, England)^57^. Western blotting was applied to detect EV markers carried out with anti-Alix (PDCD61P, Proteintech, Cat# 12422-1-AP, 1:1000), anti-TSG101 (Proteintech, Cat# 14497-1-AP, 1:1000) antibodies and negative marker, anti-Calnexin (Proteintech, Cat# 10427-2-AP, 1:1000) antibody.

### Labeling of neuronal EVs and uptake by microglia

To detect the uptake of neuronal EVs by microglia in vitro, neuronal EVs were labeled by a PKH26 Red Fluorescent Cell Linker Kit (Sigma-Aldrich, MINI26-1KT). In brief, 1.5 μL PKH26 was diluted in 100 μL diluent C, and 80 μL diluent C was added to the EVs resuspended in 20 μL PBS. Subsequently, transfer the EVs mixed with diluent C to the tube containing PKH26 in diluent C. After incubation for 5 min, the reaction was stopped by adding 100 μL PBS containing 10% EVs-free FBS (FBS was centrifuged at 140,000 × *g* for 2 h using a Ti70 rotor to deplete EVs)^58^. Then, the EVs were centrifuged at 140,000 × *g* for 2 h using an SW55Ti rotor to remove uncombined dye. After washing with PBS, the labeled EVs were resuspended. The labeled neuronal EVs were then incubated with CellTracker^TM^ Green CMFDA Dye (Invitrogen, C2925)-labeled microglia for 12 h. The addition of EVs isolated from 100 ml neuronal-conditioned media into 24-well-plate microglia culture medium was counted as concentrating 200 times (200×).

### The detection of microglial phagocytosis

Primary microglia were seeded in 24-well-plates incubated overnight in culture medium, and processed according to the pHrodo™ Red zymosan bioparticles conjugate for phagocytosis (Life Technologies, P35364) procedure. Microglia were labeled with CellTracker™ Green (1 μM Dye was added into PBS and mixed for 30 min). After twice washed with PBS, microglia were incubated with PBS containing 10 μL/ml of pHrodo Red zymosan bioparticles at 37 °C for 15 min (if microglia were treated with OGD, incubated when reoxygenation). The treated microglia were examined every 30 min by a fluorescence microscope (Zeiss, Heidelberg, Germany).

### In vitro luciferase assays

The 3′-UTRs of ptafr mRNA harboring the predicted miR-98 binding sequences were PCR amplified from human genomic DNA and cloned into Bam HI and FXho I of the pLUC-Report luciferase vector (Shenzhen Kangbio Biological Technology Co., Ltd, Shenzhen, Guangdong) to generate the ptafr-3′-UTR reporter construct. Mutagenesis of predicted targets with a mutation of 7 bp from the site of perfect complementarity was performed using a site-directed Mutagenesis Kit (Takara, 638909)^59^. HEK 293 T cells were plated at a density of 2 × 10^4^ cells/well in 96-well plates before transfection. When cells were grown to 50% confluence in 96-well plates, they were co-transfected with 0.2 μg plasmid DNA, 0.15 μg sensor reporter gene, and 0.45 μg miR-98 mimics or miRNA negative control (NC) using Lipofectamine 2000 (Invitrogen, 11668027) according to the manufacturer’s instructions. Luciferase activity was detected with a Dual-Luciferase Reporter Assay System (Promega, Cat #E1980) 48 h after transfection (using miR-101 targeting EZH2 as positive control, supplementary Fig. 3). Firefly luciferase activity was normalized to Renilla luciferase activity for each transfected well.

The primers of Ptafr-miR-98 FXho I:

5′-cacaactcgagTCATTTCCTGTGTACCGGGC-3′

The primers of Ptafr-miR-98R Bam HI:

5′-aaggatccTAAGGGACCTGCAAAGCCTG-3′

The primers of Ptafr-miR-98-MR:

5′-CTCCATCCTTTAACCTCATAGGTAATGACCCTAACTCCATCGAAATTCAGTGCCTGGT-3′

The primers of Ptafr-miR-98-MF:

5′-GATGGAGTTAGGGTCATTACCTATGAGGTTAAAGGATGGAGATGGGATTGTTATACGCC-3′.

### Drug treatment

Microglia were treated with LPS (0.01 μg/mL; Sigma-Aldrich, L2880) to establish inflammatory model in vitro. MiR-98 agomir was injected intracerebroventricularly (i.c.v) in vivo transfection technology. Entranster^TM^-in vivo transfection reagent (18668-11-1) was purchased from Engreen (Beijing, China) for in vivo, which is low toxic, efficient and no obvious inflammatory injury to animals. The in vivo transfection was performed according to the specific instructions. In brief, the negative control and rno/mmu-miR-98 agomir (1 μg/1 μL) were added to 0.5 μL of Entranster^TM^ in vivo transfection reagent. The solution was mixed gently, left for 15 min and then injected.

### Intracerebroventricular injection

For the injection of rno/mmu-miR-98 agomir, SD rats or C57BL/6 J mice were anesthetized with isoflurane and received intracerebroventricular injection using a micro syringe under the guidance of stereotaxic instruments (RWD Life Science) 3 days before tMCAO model^60^. The stereotaxic coordinates of rats (AP: −0.8 mm, ML: 1.5 mm, DV: −4.0 mm) and mice (AP: −0.8 mm, ML: 0.2 mm, DV: −3mm).

### Viral injection

pAAV-SYN-MCS-EYFP-3FLAG (9.46 × 10^12^, 0.5 μL), pAAV-SYN-CD63-mCherry-3FLAG (1.81 × 10^13^, 0.3 μL), and pAAV-SYN-EYFP-3FLAG-miR-98 (8.36 × 10^12^, 0.3 μL) purchased from Obio Technology (Shanghai, China) were unilaterally injected into cortex of P20 C57BL/6 J mice (AP: −1 mm, ML: −1.8 mm, DV: −0.2 mm). Viral titers over 1 × 10^12^ genomic particles/mL were used and injected into each location at 100 nL/min. The following experiments were performed 3 weeks after virus injection. The 24h-post-fixed brain injected with pAAV-SYN-MCS-EYFP-3FLAG was stored in 4 °C PBS and horizontally sectioned at 100 μm slices using a VT1200S Vibratome (Leica). Images were visualized and captured by a confocal microscopy (Nikon A1RSi, Tokyo, Japan) to confirm the regional expression of virus infection.

### Triphenyltetrazolium chloride staining

The infarct volume was evaluated 24 h after tMCAO. Animals were anesthetized with isoflurane, euthanized and the brains were removed. Each brain was coronally sectioned into 1 or 2 mm slices. The slices were incubated with 2% triphenyltetrazolium chloride (Sigma-Aldrich, T8877) at 37 °C for 20 min to determine the size and extent of the infarction. The pallor area indicated ischemic infarct. The brain infarct areas were analyzed with Image-Pro Plus 6.0 to estimate the infarct volume.

### Nissl staining

The paraffinized brain samples were treated with Nissl Staining Solution (Beyotime Biotechnology, C0117). A bluish–purple color was observed to display the basic nervous structure of the brain. Large and numerous Nissl bodies indicated that the nerve cells had a high ability to synthesize proteins. The number of Nissl corpuscles decreased significantly when the nerve cells were damaged. Images were captured by a fluorescence microscope (Zeiss, Heidelberg, Germany). The number of stained cells was counted from the selected fields randomly and analyzed with Image-Pro Plus 6.0.

### Western blotting

Cell samples were dissociated in 100 μL of lysis buffer consisting of 1% phenylmethanesulfonyl fluoride (KeyGEN, KGP610). The protein concentrations were determined with a BCA Protein Assay kit (Beyotime Biotechnology, P0012), and the proteins were denaturalized with 5× loading buffer. Total proteins (40 μg) were separated by sodium dodecyl sulfate—polyacrylamide gel electrophoresis and transferred to polyvinylidene fluoride membranes (Roche, 3010040001). Then, 5% skim milk in Tris-buffered saline–Tween-20 (TBST) (1 M Tris-HCl (pH 7.5), 0.8% NaCl, and 0.1% Tween-20) was used to block the membranes for 1 h at room temperature. The membranes were then incubated with the following primary antibodies at 4 °C overnight: Ptafr (abcam, Cat# ab104162, 1:200), iNOS (Santa Cruz Biotechnology, sc-7271, 1:1000), CD206 (abcam, Cat# ab64693, 1:1000), GAPDH (Proteintech, Cat# 10494-1-AP, 1:5000), α-tubulin (Proteintech, Cat# 10094-1-AP, 1:2000).

### Immunofluorescence and confocal image acquisition

Mice were anesthetized with isofluorane and perfused with PBS followed by 4% PFA. After post fixation with 4% PFA overnight, the brains were dehydrated using a graded series of alcohol, cleared in xylene, and embedded in paraffin. Then, coronal slices (5 µm) were cut by a rotary microtome (LEICA, RM2245, Heidelberg, Germany). After deparaffinization, sections of the brain were incubated in 3% H2O2 for 15 min to block endogenous peroxidases for antigen retrieval and then washed with PBS. Next, sections were incubated with 5% goat serum and 0.01% Triton-100 (dissolved in PBS) for 1 h and then stained with primary antibodies (IBA1, goat, abcam, Cat# ab5076, 1:500; IBA1, rabbit, WAKO, 019-19741, 1:500; NeuN, Millipore, MAB377, 1:400; Ptafr, abcam, Cat# ab104162, 1:200; iNOS, Santa Cruz Biotechnology, sc-7271, 1:500) overnight at 4 °C. Following washing with PBS, the sections were incubated with Alexa Fluor 488, 555, 594, and (or) 647 donkey anti-mouse, anti-goat, and (or) anti-rabbit secondary antibodies (1:1000 dilution, Invitrogen Life Technologies, NY, USA). Images were captured by a confocal microscopy or two-photon Microscope (Zeiss LSM880 with NLO & Airyscan) after incubation with 4–6-diamidino-2-phenylindole (Beyotime Biotechnology, C1005) for 15 min and washing with PBS. 3D representing of Z-stack images of neuronal EVs delivered to microglia in vivo and phagocytic uptake of neurons by microglia were acquired and 3D reconstructions were generated using ZEN software (blue/black edition). The microglial engulfment was analyzed using IMARIS software (Bitplane).

### Statistical analysis

Data were analyzed in a blinded way. Statistical analyses were performed using SPSS software, version 18.0 (SPSS Inc., Chicago, IL, USA). Multiple comparisons were conducted using one-way analysis of variance (ANOVA) and Tukey’s multiple comparisons test. Two-way ANOVA test was used when graphs are compared between brain areas or time of treatment. The means of the two treatment groups were analyzed using unpaired or paired Student’s *t* tests. Data are expressed as means±standard error of measurements. A value of *p* < 0.05 indicates that the difference was statistically significant.

## Supplementary information

Supplementary Table 1

Supplementary Fig.1

Supplementary Fig.2

Supplementary Fig.3

Supplementary Fig.4

Supplementary Fig.5

Supplementary Fig.6

Supplementary Fig.7

Supplementary Fig.8

Supplementary Figure Legends

video 1

video 2
