# Supplementary figures and images for: Neuronal extracellular vesicle derived miR-98 prevents salvageable neurons from microglial phagocytosis in acute ischemic stroke
Source: Cell Death Dis. 2021 Jan 6;12(1):23. doi: 10.1038/s41419-020-03310-2 (PMC7791117; doi:10.1038/s41419-020-03310-2)

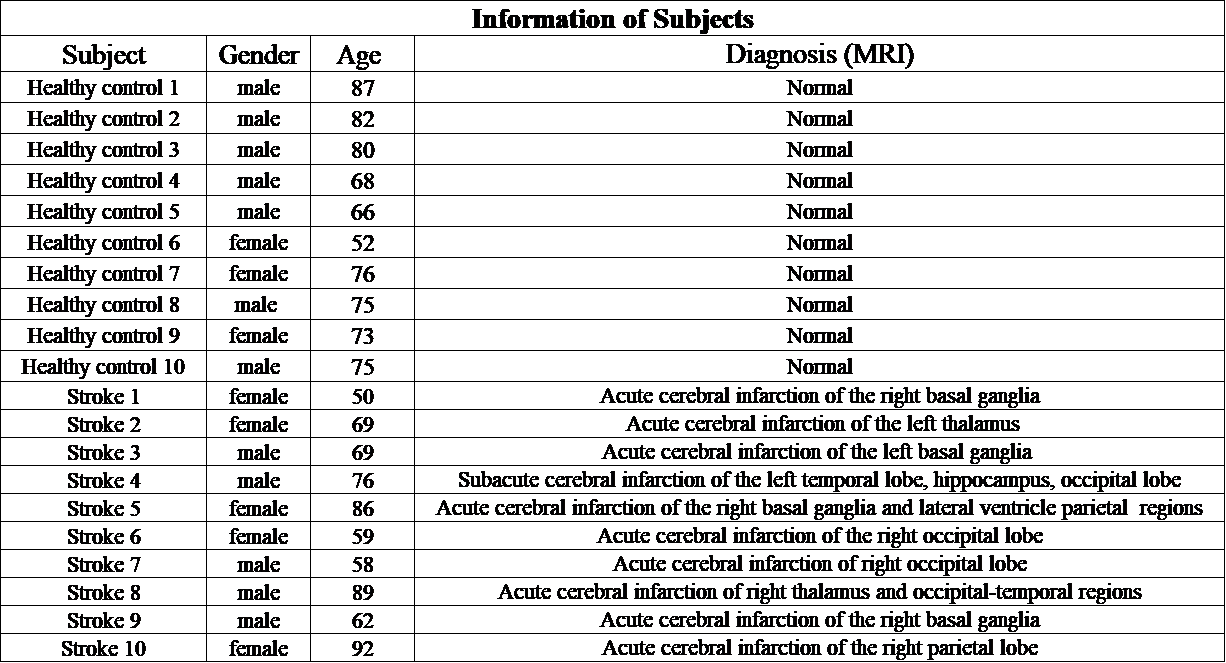

Supplement: Supplementary file 1 — Supplementary Table 1 [file 41419_2020_3310_MOESM1_ESM.png]

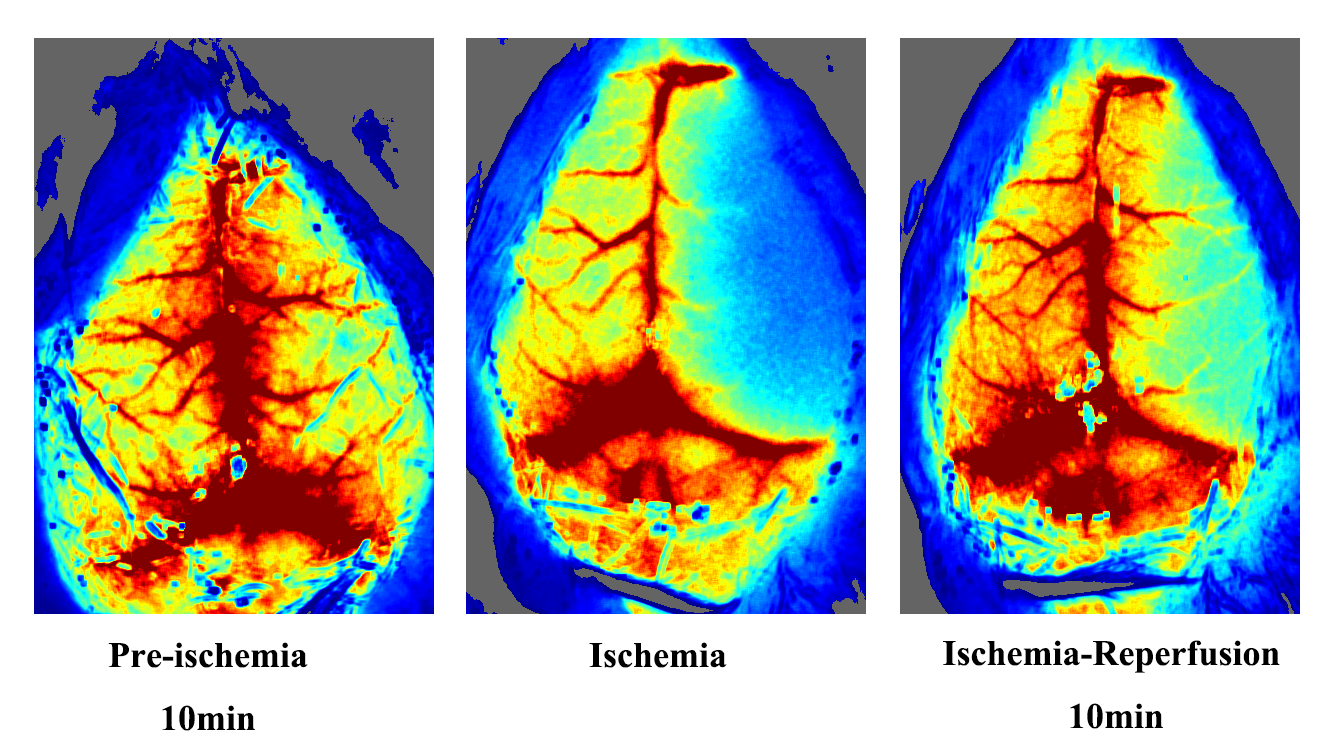

Supplement: Supplementary file 2 — Supplementary Fig.1 [file 41419_2020_3310_MOESM2_ESM.tif]

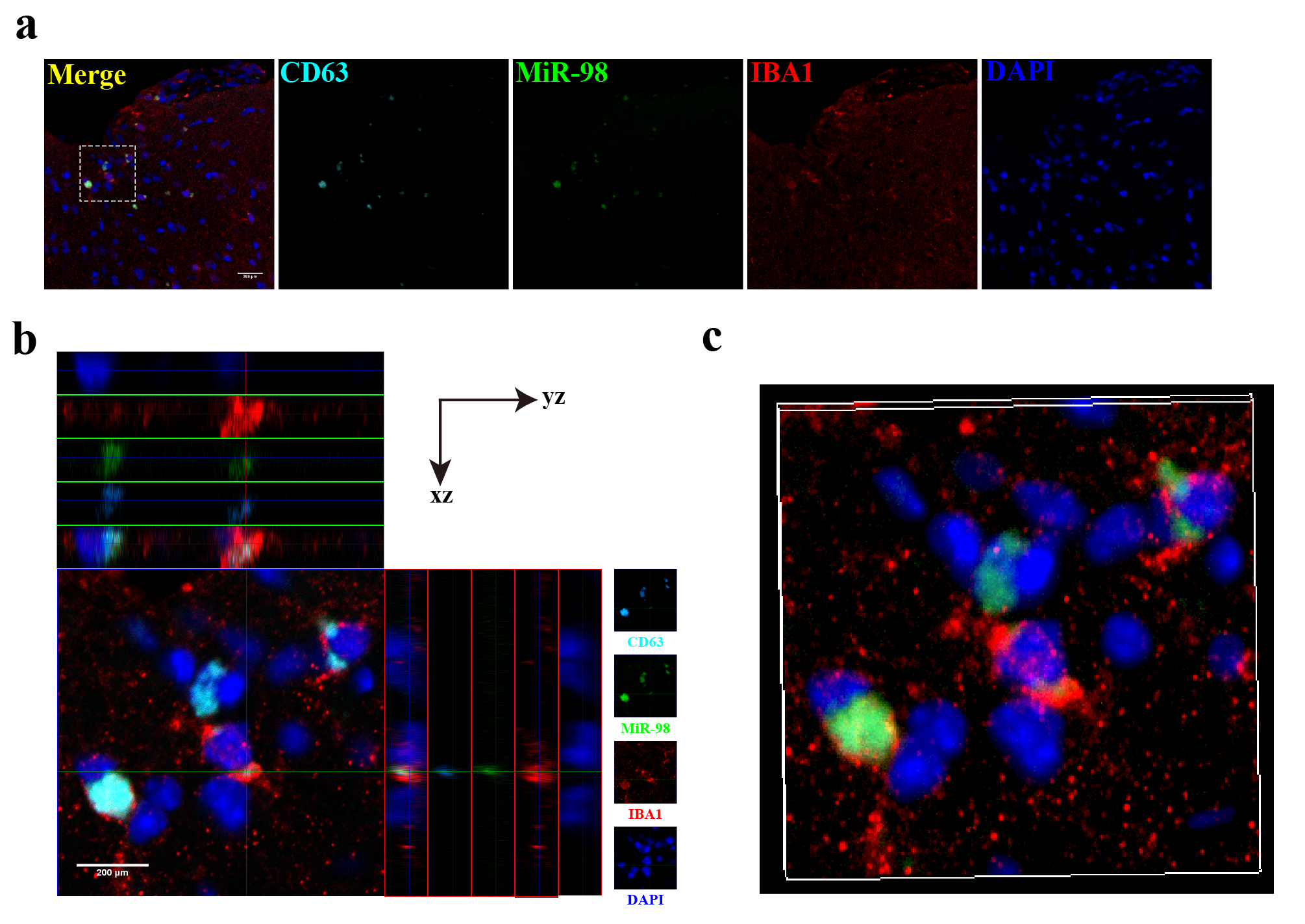

Supplement: Supplementary file 3 — Supplementary Fig.2 [file 41419_2020_3310_MOESM3_ESM.png]

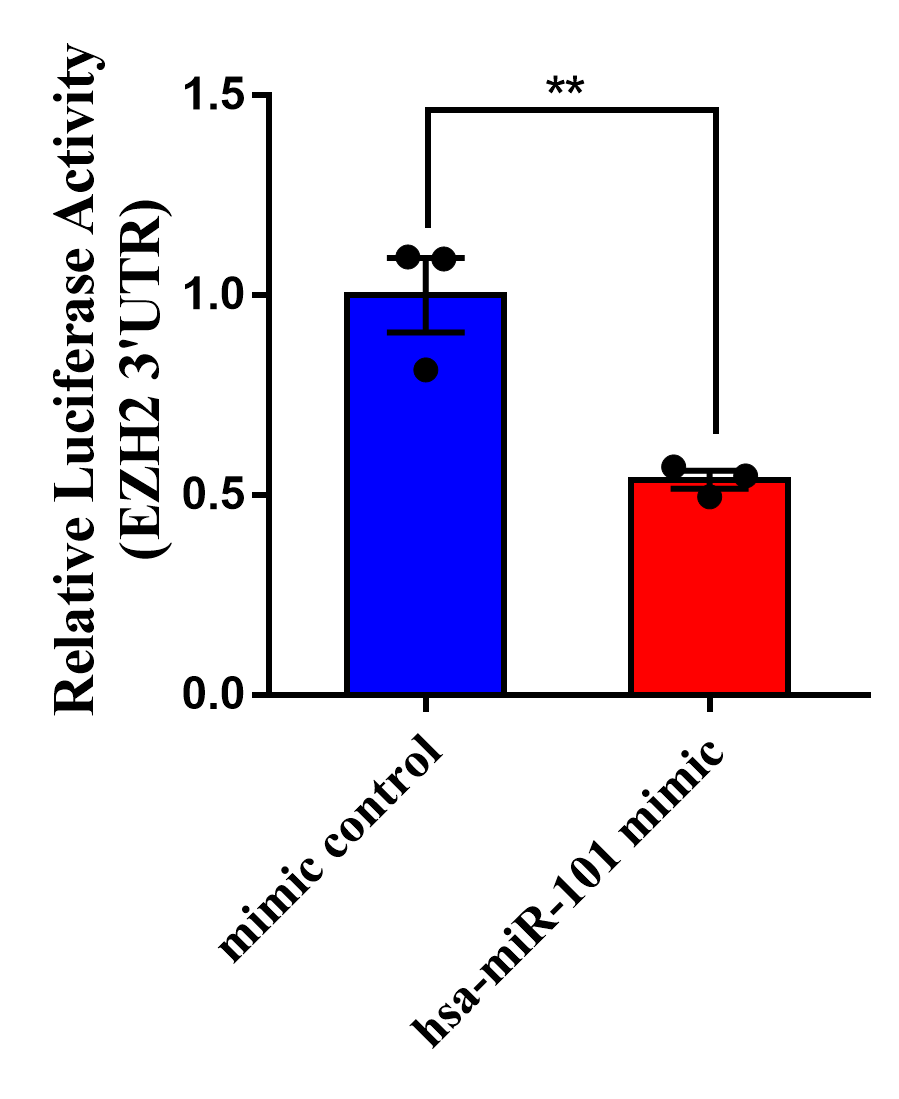

Supplement: Supplementary file 4 — Supplementary Fig.3 [file 41419_2020_3310_MOESM4_ESM.tif]

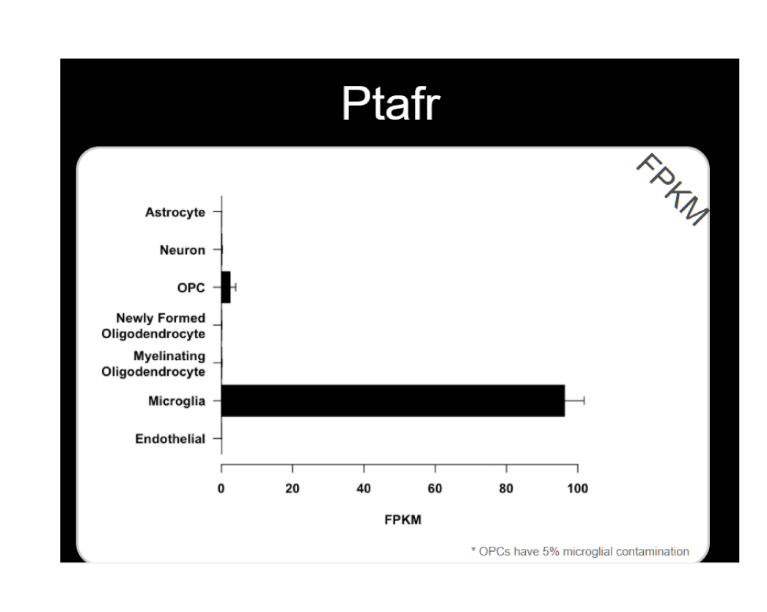

Supplement: Supplementary file 5 — Supplementary Fig.4 [file 41419_2020_3310_MOESM5_ESM.tif]

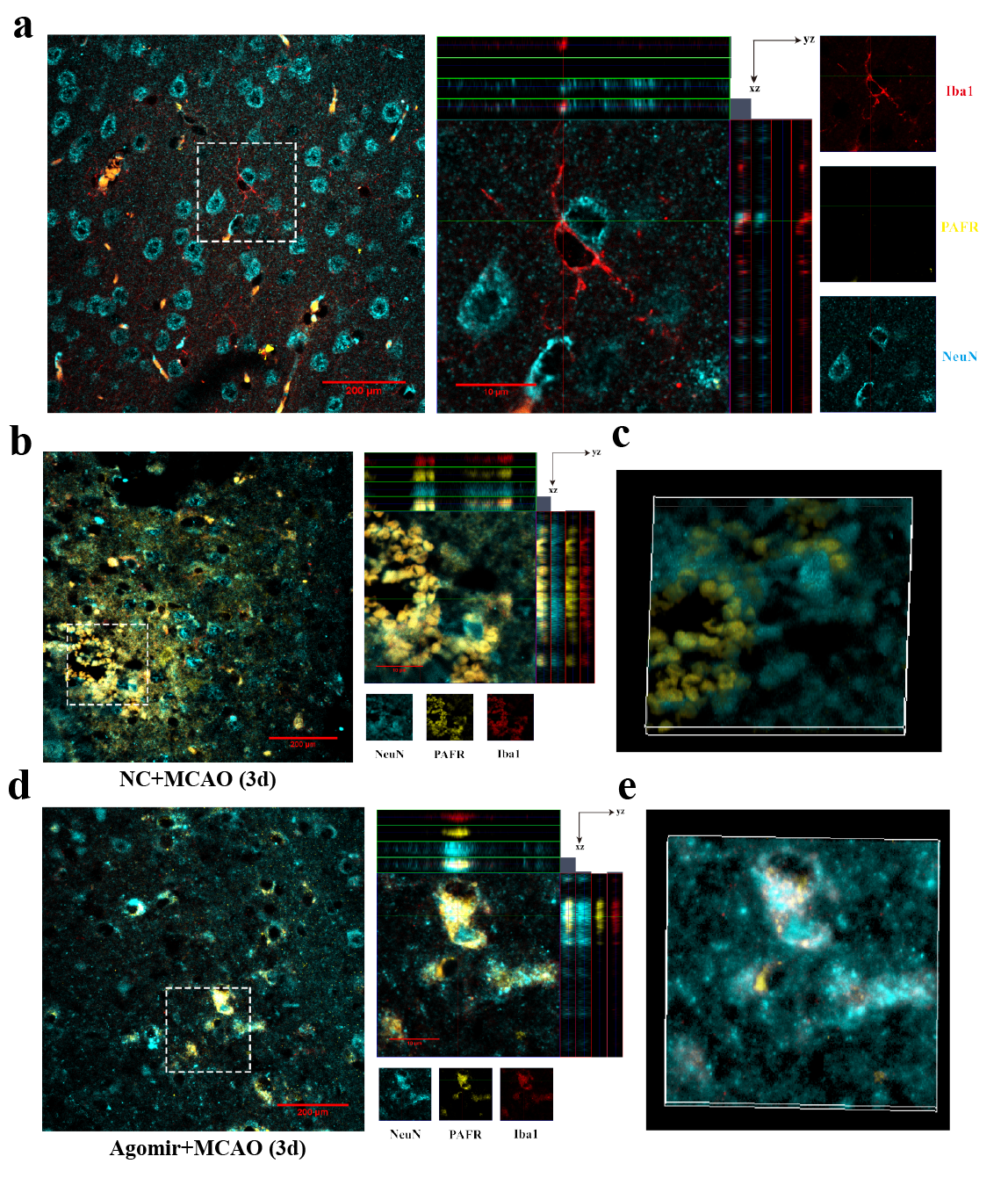

Supplement: Supplementary file 6 — Supplementary Fig.5 [file 41419_2020_3310_MOESM6_ESM.png]

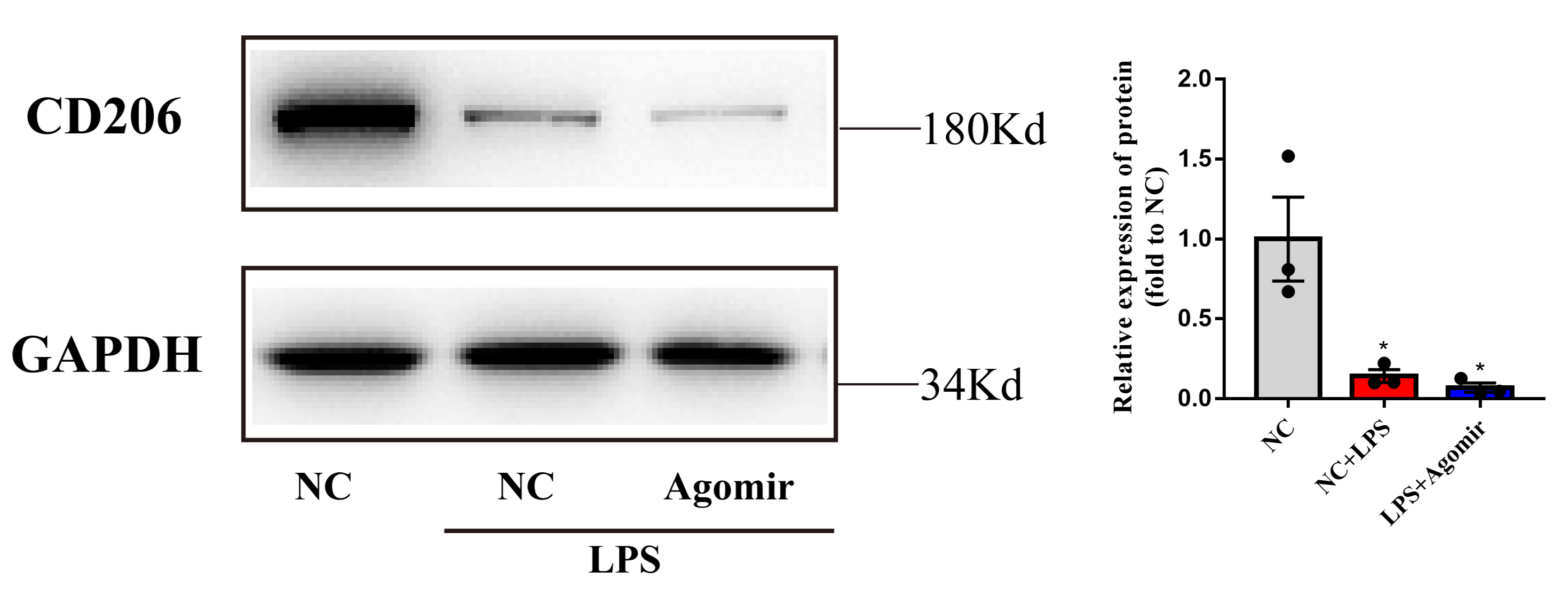

Supplement: Supplementary file 7 — Supplementary Fig.6 [file 41419_2020_3310_MOESM7_ESM.tif]

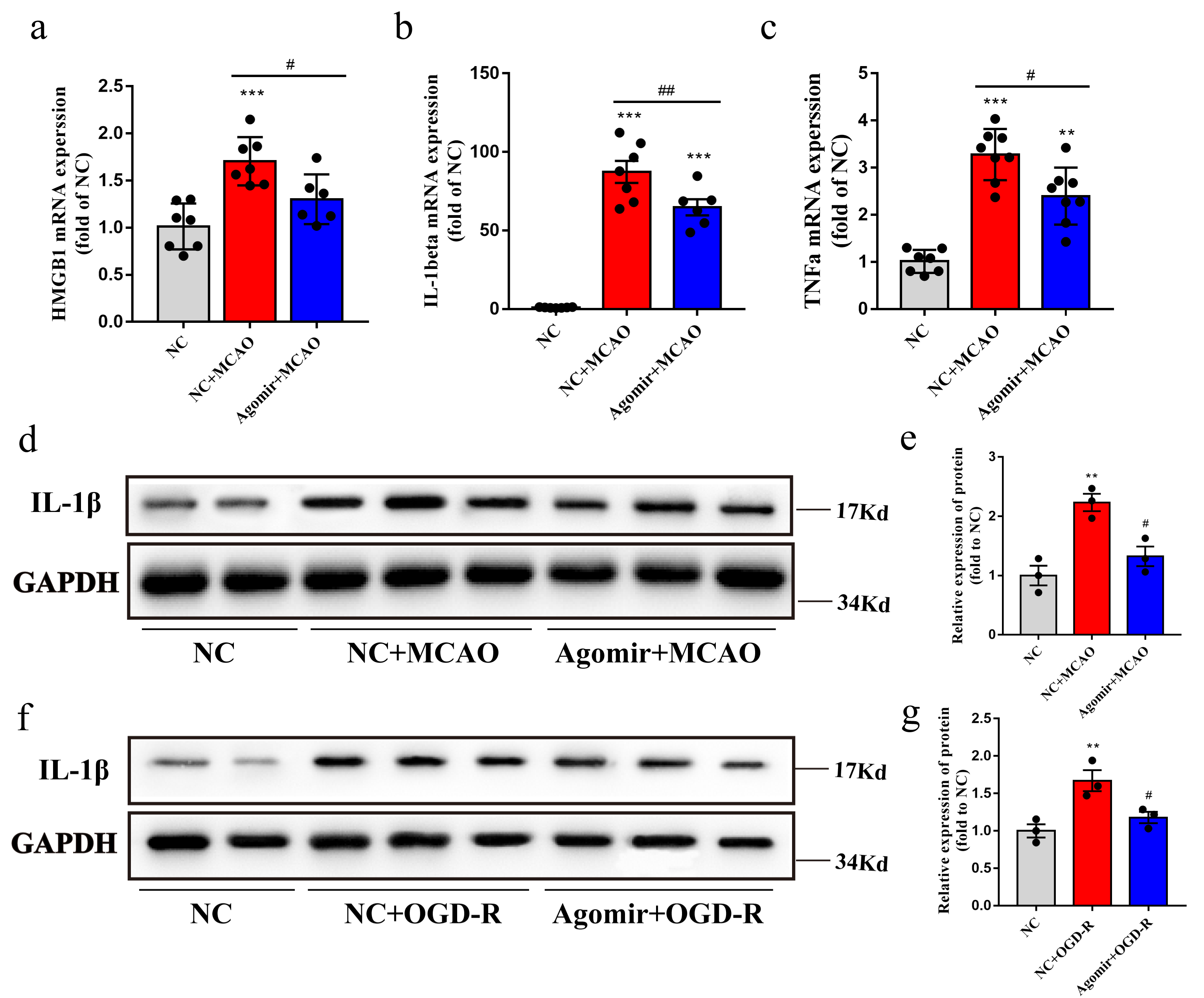

Supplement: Supplementary file 8 — Supplementary Fig.7 [file 41419_2020_3310_MOESM8_ESM.tif]

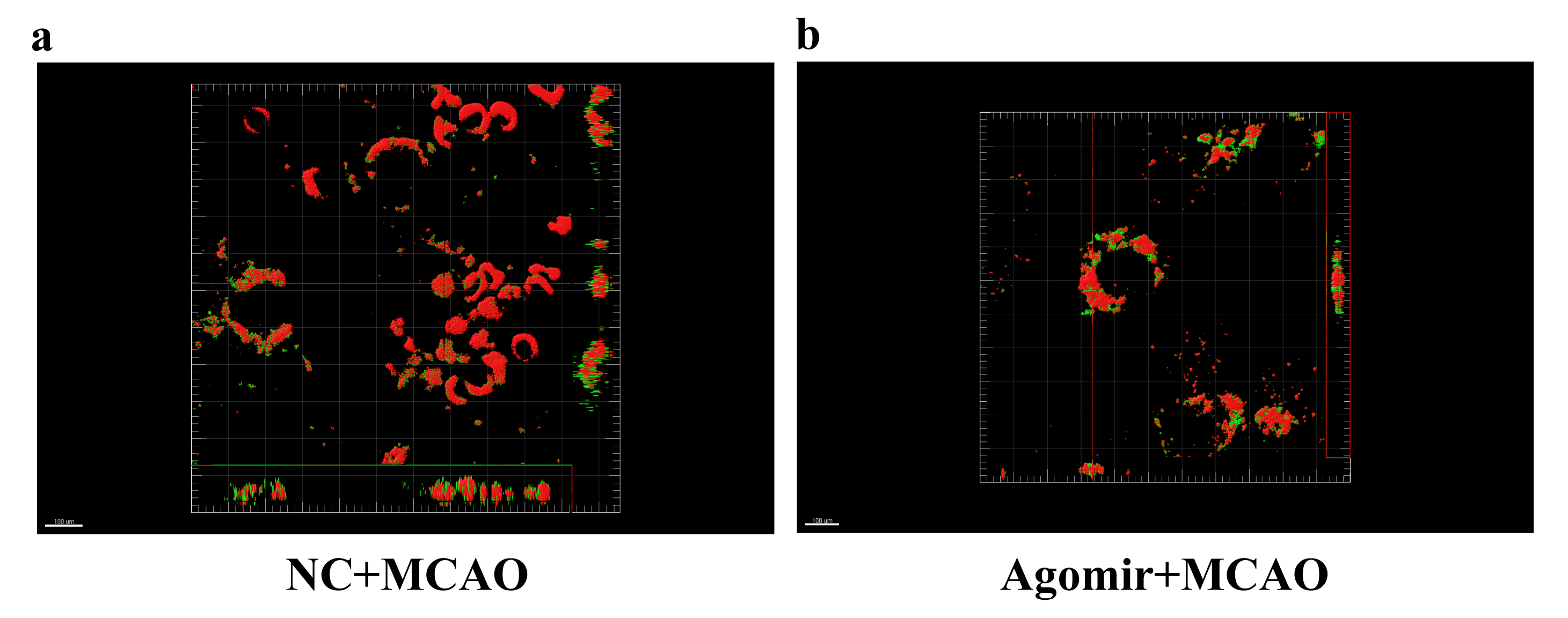

Supplement: Supplementary file 9 — Supplementary Fig.8 [file 41419_2020_3310_MOESM9_ESM.tif]
