# Supplementary material for: Neuronal extracellular vesicle derived miR-98 prevents salvageable neurons from microglial phagocytosis in acute ischemic stroke
Source: Cell Death Dis. 2021 Jan 6;12(1):23. doi: 10.1038/s41419-020-03310-2 (PMC7791117; doi:10.1038/s41419-020-03310-2)
Supplement: Supplementary file 10 — Supplementary Figure Legends [file 41419_2020_3310_MOESM10_ESM.docx]

**Supplementary Figure legends**

**Supplementary Table 1. The information of human subjects.**

**Supplementary Fig. 1** The blood flow changes identification in tMCAO model.

**Supplementary Fig. 2**

(a-c) The representative confocal images, amplification and colocalization analysis, and 3D reconstructions of CD63-mCherry co-labeled with miR-98-EYFP and IBA1 on paraffin slices 3 h after ischemic stroke acquired by a confocal microscopy. (5 μm thickness, Scale bar = 200 μm). (n=3 independent experiments)

**Supplementary Fig. 3**

The positive control of dual luciferase reporter gene assay (miR-101)

**Supplementary Fig. 4**

The prediction of PAFR transcriptional expression in the database of cell type expression categories in the cerebral cortex of mice. FPKM: Fragments per kilobase of transcript per million mapped reads. (Zhang Y et al (2014) An RNA-sequencing transcriptome and splicing database of glia, neurons, and vascular cells of the cerebral cortex. The Journal of neuroscience: the official journal of the Society for Neuroscience 34: 11929-11947 Doi 10.1523/jneurosci.1860-14.2014)

**Supplementary Fig. 5**

(a) The representative immunostainings and Z-stack images of contralateral NeuN, IBA1, and PAFR colocalization in mice brain slices after ischemic stroke. (b-e) The representative immunostainings and Z-stack images, 3D reconstructions of NeuN, IBA1, and PAFR colocalization in mice brain slices of mice with NC/miR-98 agomir treatment 3d after ischemic stroke. (another example)

**Supplementary Fig. 6**

The protein expression level of CD206 and quantification. (n=3 independent experiments)

**Supplementary Fig.7**

(a-c) The mRNA expression level of HMGB1, IL-1β, TNF-α in tissues. (n=7 independent experiments) (d-g) The protein expression level of IL-1β and quantification in tissues and microglia. (n=3 independent experiments)

**Supplementary Fig.8**

（a-b）The quantification of the phagocytosis of microglia using IMARIS software (Bitplane). Reference: Schafer, D.P., Lehrman, E.K., Heller, C.T., Stevens, B. An Engulfment Assay: A Protocol to Assess Interactions Between CNS Phagocytes and Neurons. J. Vis. Exp. (88), e51482, doi:10.3791/51482 (2014).
